# Self-trainable and adaptive sensor intelligence for selective data generation

**DOI:** 10.3389/frai.2024.1403187

**Published:** 2025-01-22

**Authors:** Arghavan Rezvani, Wenjun Huang, Hanning Chen, Yang Ni, Mohsen Imani

**Affiliations:** Donald Bren School of Information and Computer Sciences, University of California, Irvine, Irvine, CA, United States

**Keywords:** active learning, intelligent sensing, Internet of Things, knowledge distillation, machine learning, near-sensor computing

## Abstract

With the increasing integration of machine learning into IoT devices, managing energy consumption and data transmission has become a critical challenge. Many IoT applications depend on complex computations performed on server-side infrastructure, necessitating efficient methods to reduce unnecessary data transmission. One promising solution involves deploying compact machine learning models near sensors, enabling intelligent identification and transmission of only relevant data frames. However, existing near-sensor models lack adaptability, as they require extensive pre-training and are often rigidly configured prior to deployment. This paper proposes a novel framework that fuses online learning, active learning, and knowledge distillation to enable adaptive, resource-efficient near-sensor intelligence. Our approach allows near-sensor models to dynamically fine-tune their parameters post-deployment using online learning, eliminating the need for extensive pre-labeling and training. Through a sequential training and execution process, the framework achieves continuous adaptability without prior knowledge of the deployment environment. To enhance performance while preserving model efficiency, we integrate knowledge distillation, enabling the transfer of critical insights from a larger teacher model to a compact student model. Additionally, active learning reduces the required training data while maintaining competitive performance. We validated our framework on both benchmark data from the MS COCO dataset and in a simulated IoT environment. The results demonstrate significant improvements in energy efficiency and data transmission optimization, highlighting the practical applicability of our method in real-world IoT scenarios.

## 1 Introduction

Over the past few years, the Internet of Things (IoT) has garnered substantial research interest. The commonly recognized definition of the IoT characterizes it as a network infrastructure that enables the connection of various objects to the Internet using specified protocols (Patel et al., 2016). This connectivity allows for information sensing equipment to facilitate the exchange and communication of data, enabling smart recognition (Bianchi et al., 2019), positioning (Ghazal et al., 2021), monitoring (Huang et al., 2021), and administration capabilities (Kim et al., 2017). According to a recent forecast by the International Data Corporation, the estimated number of IoT devices will reach 55.7 billion by 2025. Meanwhile, these devices are expected to generate ~80 zettabytes of data. This exponential growth in devices and data poses significant challenges for real-time processing and analysis in IoT ecosystems. To handle this deluge of data, many IoT applications rely on complex machine learning (ML) models to analyze sensor-collected data (Afshan and Rout, 2021; Mahdavinejad et al., 2018; Yang and Shami, 2022; Ha et al., 2020; Sharma et al., 2022; Huang et al., 2024a).

However, despite ML models empowering sensing frameworks with the capability to execute complex tasks [e.g., classification (Lin et al., 2020), segmentation (Wan et al., 2022), pose estimation (Huang et al., 2024b)], they face significant challenges during actual deployment in real-world IoT environments. Due to the constrained computational capabilities of the edge devices, deployed ML models experience difficulties concerning energy consumption, inference speed, and accuracy (Li and Pŕıncipe, 2021). These are especially problematic for applications that require a relatively complex and expensive ML model. A common approach to address this limitation is to offload computationally expensive tasks to more powerful central servers (Huang et al., 2019). Nevertheless, naive offloading the tasks to a central server results in considerable extra resource pressure and wastage since it lacks targeted intelligence (Tsakanikas et al., 2023). In many IoT applications [e.g., fire alarm, crime surveillance (Yogameena et al., 2019), wildlife monitoring], only a small fraction of data generated by sensors contains useful information. Therefore, continuously running computationally expensive ML models on dense, redundant data is both resource-intensive and inefficient. It leads to a continuous process using complex ML models on dense data, while only a small fraction of frames carry out useful information. In fact, the ML model only targets that small fraction of data, but it still has to process large amounts of unnecessary data. To mitigate these inefficiencies, researchers have proposed several alternative approaches. One method involves compressing sensor data before transmission (Redondi et al., 2013). While compression can reduce energy and storage consumption, it often introduces trade-offs. Mild compression may still accumulate substantial data over time, whereas aggressive compression can degrade data quality, adversely impacting downstream analyses (Bagdanov et al., 2011; Tsifouti et al., 2012). Another approach involves transmitting extracted features from the raw data (Redondi et al., 2013), utilizing feature extraction techniques such as SIFT (Lowe, 2004), SURF (Bay et al., 2006), or BRISK (Leutenegger et al., 2011). However, these methods often lack generalization, as the extracted features are highly task-specific, limiting their versatility for broader analytical purposes.

Intelligent sensing frameworks, such as the one proposed in Huang et al. (2024c), provide a promising solution to address energy consumption challenges in IoT systems. By selectively transmitting only sparse and relevant data—referred to as Frames of Interest (FoI)—these frameworks significantly reduce unnecessary data transmission while maintaining the quality of transmitted frames. This is achieved through a lightweight ML model deployed near the sensor, which identifies FoI for further processing by a more powerful central server. Inspired by biological sensors that generate data volumes orders of magnitude smaller than the raw sensed input (Dodda et al., 2022), intelligent sensing offers a practical means to reduce resource consumption in IoT applications. However, the seamless integration of intelligent sensing into conventional sensing frameworks remains challenging. Current intelligent sensing frameworks rely on pre-trained lightweight models near the sensor, which lack adaptability and generalizability compared to more complex central models. Consequently, their inference performance is heavily influenced by variations in input data distributions, limiting their effectiveness in dynamic real-world environments.

In this study, we propose a novel approach to overcome these limitations by incorporating online learning into near-sensor models. Unlike existing methods, our approach enables intelligence to be integrated into conventional sensing systems without requiring costly manual labeling or pre-training. By leveraging online learning, the near-sensor model can adapt dynamically to environmental variations and fine-tune itself based on the predictions of a more complex central model. This results in the development of an adaptive intelligent sensing framework that is both flexible and responsive to changes in data distributions. To further enhance efficiency, we integrate the following key techniques into the framework: (1) Active Learning (AL): reduces energy consumption and storage requirements by selectively identifying the most informative data points for training the near-sensor model. (2) Knowledge Distillation (KD): compresses the near-sensor model using the knowledge from the central model, significantly reducing its size and computational complexity while maintaining performance.

By combining these components, our framework introduces a novel, energy-efficient, and self-adaptive intelligent sensing approach that enables near-sensor models to continuously learn and optimize their performance post-deployment.

The key contributions of this work are as follows:

- Unsupervised near-sensor training: we demonstrate that the near-sensor model can be trained without any manual labeling, leveraging predictions from the central model and environmental data collected post-deployment.
- Continuous adaptation: the system is designed to automatically learn and adjust to dynamic environmental changes, enabling long-term adaptability in real-world IoT scenarios.
- Zero pre-training: the intelligent sensing framework operates without initial pre-training of the near-sensor model, reducing setup costs and improving flexibility.
- Model compression via KD: knowledge distillation effectively compresses the edge model, reducing its resource demands while maintaining high accuracy.
- Energy-efficient training: we develop strategies for training the near-sensor model under strict energy and data constraints, addressing practical limitations in IoT environments.

The proposed framework is a comprehensive solution for achieving energy-efficient, adaptable, and intelligent sensing in IoT systems. The subsequent sections provide a detailed exploration of each component and its integration within the framework.

## 2 Related works

### 2.1 Intelligent sensing

Previous work in Huang et al. (2024c) presents the idea of intelligent selective data transmission in sensing frameworks, which we refer to as intelligent sensing for short, and forms the core foundation of our current work. This intelligent sensing framework involves the deployment of a lightweight machine learning model near the sensor. The near-sensor model is designed for the detection of FoIs. The authors utilized the YOLOv5 model family as their near-sensor model, which is a well-known object detection model. This model is capable of detecting objects of interest at any scale and in any location inside the frame. The YOLOv5 model computes objectness scores for each frame and transmits frames that surpass a predefined threshold, ensuring that only frames containing the object of interest are transmitted.

Since the YOLOv5 model is deployed near the sensor, it needs to be as lightweight as possible. To address this requirement, the authors proposed reducing both the width and depth of the YOLOv5n (nano), which is the smallest variant of the YOLOv5 model. As a result, they were able to create two additional models: YOLOv5nm with half the depth and width of YOLOv5n, and YOLOv5ns with one-third of the depth and width of YOLOv5n. In addition, they introduced a customized loss function, which places a stronger emphasis on the objectness score rather than precise bounding box prediction. This adjustment was made to facilitate faster convergence and enhance performance compared to the original loss function in this particular context.

**Figure 1:**
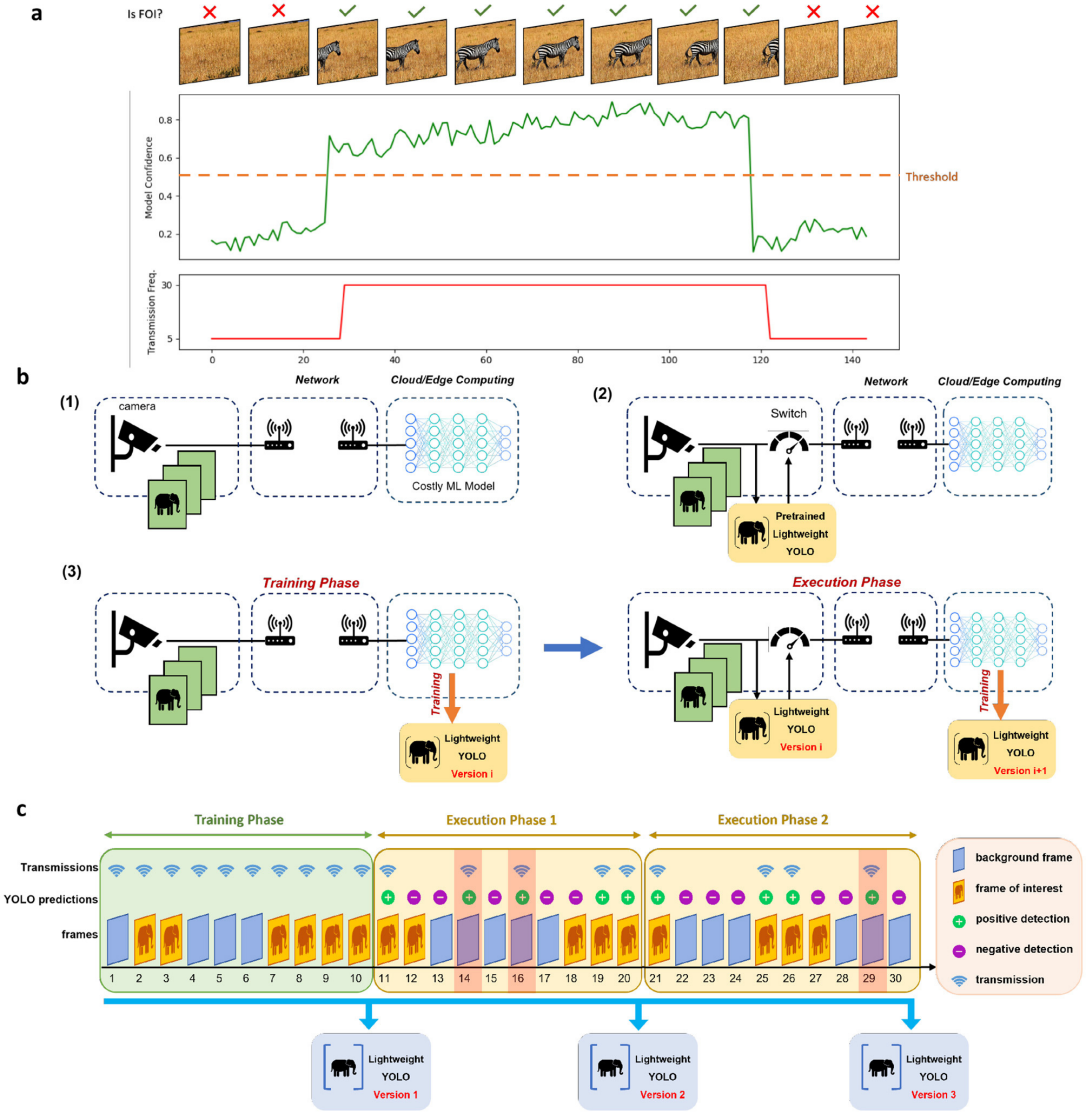
Sensing frameworks. **(A)** Intelligent sensing framework exploits the confidence of the near-sensor model to detect FoIs and tune its transmission frequency. **(B)** Comparison of conventional sensing (1), intelligent sensing (2), and the adaptive intelligent sensing (3) frameworks. The conventional sensing framework transmits all the frames captured by the sensor, while the intelligent sensing framework utilizes a pretrained lightweight model near the sensor to only transmit frames containing valuable information. The general intelligent sensing framework does not require the pretrained model from the beginning, but it enables the training of the lightweight model only based on the predictions of the server-side model. **(C)** The adaptive intelligent sensing framework exploits a training phase and multiple execution phases. In the training phase, all the frames are transmitted to the server. In the execution phase, the near-sensor model is exploited to extract and transmit the positive frames. From these transmissions of the execution phase, the misdetections are stored on the server. After each phase, the near-sensor model will be updated based on the stored frames. It's worth mentioning that in this figure, FoI and background frames correspond to the positive and negative detections of the server-side model. The highlighted frames (frames 14, 16, and 29) in the execution phases are the transmitted frame in which the YOLO and server-side model do not agree on the labels, thus considered as misdetections, and are added to the near-sensor model's training set.

The near-sensor model is subsequently employed to toggle a switch on and off based on its detections. When the near-sensor model detects an FoI, the sensor transmits the captured frames at full resolution and at the camera's refresh rate. However, when no FoI is detected, the transmission frequency is reduced to zero. To mitigate potential misdetections and information loss at the server, the authors propose the adoption of a predetermined non-zero minimum transmission frequency for non-FoIs. Their experiments validate the enhanced performance resulting from this approach. Figure 1A represents how an intelligent sensor utilizing a non-zero minimum transmission frequency exploits the near-sensor model's confidence to set the transmission frequency. Additionally, they introduce a “lazy sensor deactivation” scheme, which leverages the temporal correlation among frames, gradually reducing the transmission frequency to alleviate potential misdetections.

### 2.2 Knowledge distillation

Knowledge distillation (KD) is a method to transfer knowledge from a larger network or ensemble of networks (teacher model) to a smaller and less complex model (student model) (Hinton et al., 2015). It can be considered as a way to compress a larger model into a smaller one, making it more efficient and less resource-hungry, which is most effective for deploying models on edge devices (Gou et al., 2021). The intuition behind KD is that supervising the student model with the teacher model helps the student model to mimic the teacher model with comparable accuracy. The distilled knowledge from the teacher model also reveals some underlying patterns in the data, making it easier to learn by a smaller model. The very first idea of knowledge distillation is presented in Buciluǎ et al. (2006), where the student model utilizes the predictions of the teacher model on a large set of pseudo data (unlabeled or synthetic data with the same distribution as the original training data) to get an idea of the function learned by the teacher model. The idea is generalized in Hinton et al. (2015) by formalizing knowledge distillation as a method to supervise a small student by a large teacher model to obtain a competitive performance.

## 3 Method

Our proposed framework builds upon the previously introduced intelligent sensing framework (Huang et al., 2024c), enhancing its adaptability and efficiency through the integration of online learning, active learning, and knowledge distillation. In Huang et al. (2024c), the near-sensor model is tasked with identifying FoI. While this is conceptually similar to a classification task, object detection is more suitable for practical sensing scenarios, as objects of interest may vary in scale and location within the frame. Furthermore, identifying the location of objects in the frame can facilitate downstream tasks on the server side, improving overall processing efficiency. To address these requirements, we utilize a customized YOLOv5 model as the near-sensor model. YOLOv5's balance of computational efficiency and high detection accuracy makes it well-suited for resource-constrained IoT environments. The integration of YOLOv5 enables the near-sensor model to not only identify relevant frames but also extract detailed spatial information about objects of interest, further optimizing the efficiency of data transmission and server-side analysis. Figure 1B illustrates how the general intelligent sensing framework is enhanced by incorporating these intelligent and adaptive features.

The following sections detail the key methodologies employed in the adaptive intelligent sensing framework: online learning for near-sensor model adaptability, knowledge distillation for model efficiency, and active learning for resource-constrained training data selection.

### 3.1 Online learning

To empower intelligence in a sensing framework, a lightweight model is deployed near the sensor to detect Frames of Interest (FoI). Only the FoI are transmitted to the server for further analysis. Traditionally, the near-sensor model must be pre-trained on a labeled dataset, which imposes significant costs for data labeling, especially in conventional sensing frameworks. This requirement creates a barrier to seamlessly upgrading existing sensing systems into intelligent ones.

Our adaptive intelligent sensing framework addresses this limitation by leveraging online learning to train the near-sensor model post-deployment. In this framework, the near-sensor model can be deployed without pre-training and incrementally trained using the predictions of a complex server-side ML model. These predictions serve as pseudo-ground-truth labels for the near-sensor model. This approach offers two major benefits: (1) Eliminates manual labeling costs: since the complex server-side model generates pseudo-labels, there is no need for costly manual annotation. (2) Optimized near-sensor accuracy: the near-sensor model aims to replicate the server-side model's predictions, ensuring consistency between their outputs. While the near-sensor model may not surpass the accuracy of the server-side model, this is by design: the near-sensor model functions primarily as a filter, and the server-side model ultimately determines the final output.

Even if the near-sensor model detects an FoI that the server cannot process, the server disregards it, ensuring that the system's overall accuracy is not compromised. Consequently, using server-side predictions as ground truth is both effective and efficient for training the near-sensor model. Figure 1C illustrates the adaptive intelligent sensing framework. In this framework, FoI and background frames are defined based on the predictions of the complex server-side model. The framework operates in two distinct phases:

1. Training phase: in this phase, the system functions like a conventional sensing framework, transmitting all captured frames to the server. On the server side, all frames and their corresponding predictions from the complex model are stored. The stored data is used to train or retrain the near-sensor model. To minimize energy consumption on edge devices, training occurs server-side. Once training is complete, the updated model weights are transmitted back to the sensor.
2. Execution phase: during this phase, the sensing framework transmits frames based on the predictions of the near-sensor model. Misdetections (discrepancies between the predictions of the near-sensor model and the complex model) are inevitable. These misdetections are stored on the server for retraining the near-sensor model. At the end of the execution phase, the near-sensor model is retrained using a dataset comprising all frames collected during previous training phases and misdetections from prior execution phases.

The system alternates between training and execution phases, adapting dynamically to environmental changes. This iterative process ensures that the near-sensor model continuously learns and improves while adhering to energy constraints.

### 3.2 Data selection via active learning

The consecutive training and execution phases in our framework require storage and computational resources. However, in scenarios where resource limitations exist—such as constrained storage, energy, or time—retaining all sensor-collected data for retraining the near-sensor model is infeasible. To address these challenges, we introduce a data selection mechanism based on AL. The goal is to store only a subset of data that preserves the model's performance while adhering to resource constraints.

AL reduces the need for labeled data by focusing on the most informative or uncertain samples (Settles, 2009). In our framework, we define a buffer with a fixed capacity, representing the maximum number of frames that can be stored on the server for training. At the end of each phase, the buffer is refined to ensure it contains the most valuable data for retraining. The refinement process differs depending on whether the phase is a Training phase or an Execution phase, as described below.

#### 3.2.1 Training phase

For refining the buffer at the end of a training phase, where we have transmitted all the sensor-collected data to the server, first, half of the capacity of the buffer is dedicated to previous samples; meaning that, we chose at random half of the frames stored in the buffer from the previous phases and discarded them. The rest of the buffer is filled with uncertain samples from the sensor-transmissions of this phase. To this end, we follow these steps:

1. Feed all the sensor-transmitted data to the trained near-sensor model.
2. For each frame, there will be many bounding box predictions with their corresponding model confidence. Keep the maximum confidence for each frame.
3. Sort the maximum confidence values from the previous step.
4. Define a threshold of objectness, meaning that, if the maximum confidence of an image is below that threshold, we consider no objects in that image. We define such a threshold in order to keep the most informative data from our training phase.
5. Remove frames with the maximum confidence below the defined threshold.
6. Keep the frames corresponding to the least confidence values until the buffer is filled.

**Figure 2:**
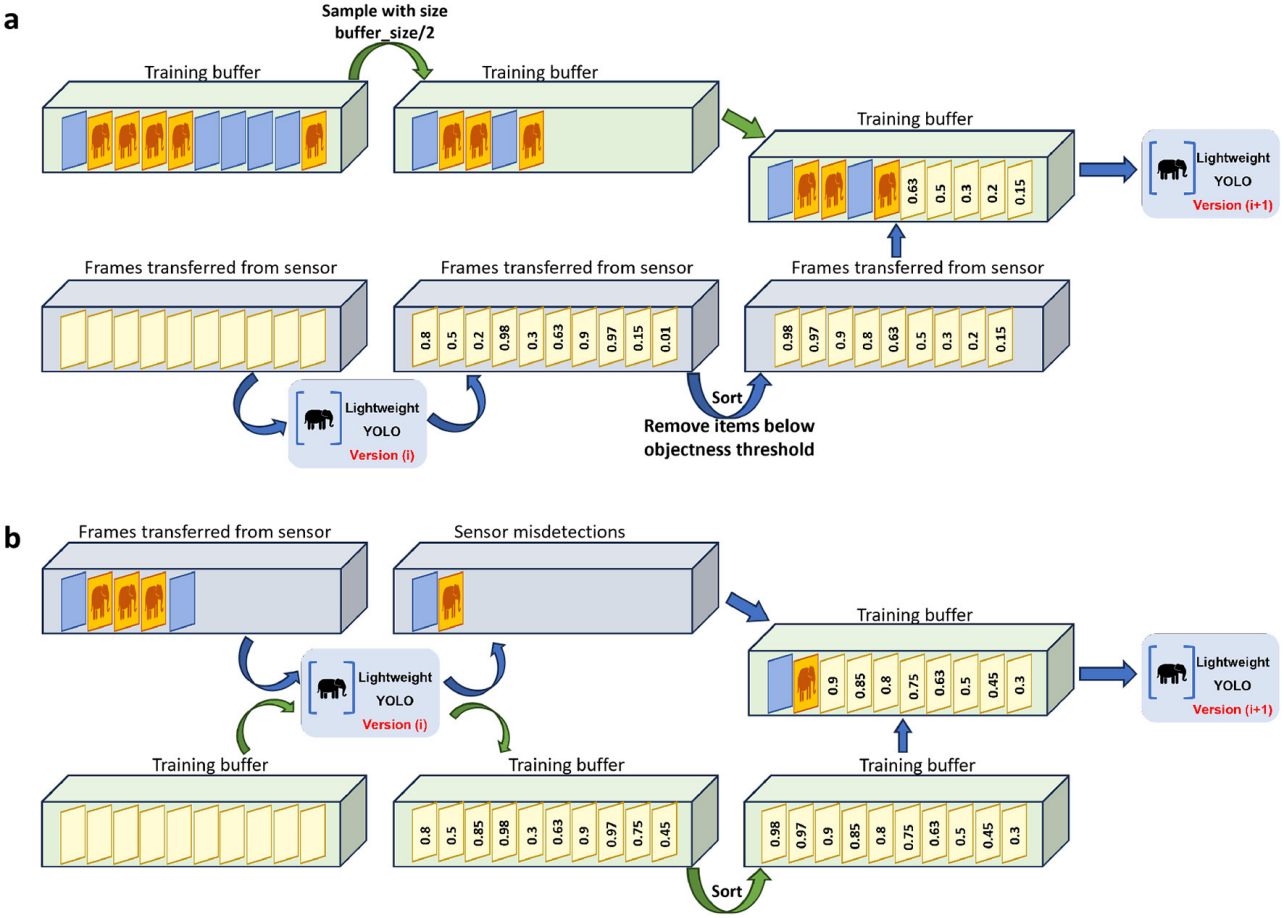
Utilizing active learning empowers our model to overcome resource constraints. The training buffer in this case has a limited capacity, and at the end of each phase must be refined in order to keep the most useful information in the buffer for training. **(A)** At the end of each training phase, half of the buffer is dedicated to previous samples, and we remove at random half of the samples in the buffer. Then we get the near-sensor model prediction on the sensor-transmitted frames, remove those with confidence value below the objections threshold, and fill the rest of the buffer with the most uncertain samples. **(B)** In the Execution phase, the sensor transmitted data contains misdetections and also correctly detected samples, which we only need to keep the misdetections, therefore we sort all the previous training buffer samples based on their confidence values, and then remove the most certain ones to let the misdetections be added to the training buffer.

This combination is beneficial for model training; the first half of the previous samples chosen at random can be beneficial for the model to remember the general pattern of the data. The reason is that only relying on the uncertain samples may mislead the model. Moreover, relying only on the learned samples disables the opportunity to improve model performance on the recently captured data. Therefore a combination of both would bring best of both worlds for near-sensor model training. Figure 2A visualizes the buffer design during the training phase.

#### 3.2.2 Execution phase

In the execution phase, only a small fraction of the sensor-captured data is transmitted to the server due to the filtering effect of the near-sensor model. At the end of the phase, the buffer is refined by focusing exclusively on misdetections (cases where the near-sensor model's predictions differ from the server-side model). These misdetections are critical for retraining, as they highlight areas where the near-sensor model needs improvement. The steps for refining the buffer during the execution phase are as follows:

1. Use the trained near-sensor model to evaluate frames stored in the training buffer.
2. Compare the near-sensor model's predictions with those of the server-side model: We retain only misdetections (frames where the two models disagree). And we discard frames with high confidence predictions, as these are considered less informative for retraining.
3. Replace the most certain samples in the buffer with newly identified misdetections.

This approach ensures that the buffer focuses on samples that are most likely to improve the near-sensor model's performance. Since the near-sensor model's accuracy improves with each phase, the number of misdetections is expected to decrease over time. Removing the most certain samples from the buffer is unlikely to impact training negatively, as these samples contribute less to refining the model. Figure 2B visualizes the buffer design during the execution phase.

### 3.3 Near-sensor model compression using knowledge distillation

To enhance the efficiency of the near-sensor model in this study, we employ KD by transferring knowledge from a trained near-sensor model to a compact one. Both models belong to the YOLO family. Typically, in object detection models, the imbalance between foreground and background instances, coupled with the simultaneous requirements for localization and classification, hinders the effectiveness of KD methods originally designed for classification tasks. Moreover, object detection models prioritize local regions that overlap with ground truth objects. Consequently, minimizing the discrepancy between the full feature maps of the teacher and student models introduces substantial noise from regions that are less relevant.

Taking into account these considerations, we applied the KD method introduced in Wang et al. (2019) to train the student YOLO model. This involved utilizing ground truth labels (in our study, derived from the output of the complex model on the server) and imitating the teacher's feature response on close object anchor locations. This approach is particularly valuable in situations where there are strict constraints on the near-sensor model. By training a compact model using knowledge from a larger one, we can achieve improved performance compared to training the smaller model independently, without assistance from the larger model. To enhance the performance, we adopted the approach suggested in Huang et al. (2024c) to adjust the student model's loss function. Specifically, we modified it to consider only the objectness score, excluding the bounding box terms. Despite the teacher model not being trained with this modified loss function, applying it to the student model has been shown to significantly improve its performance. This approach effectively reduces the size of the near-sensor model while preserving its performance at a comparable level.

## 4 Results

### 4.1 Experimental setup

In this study, our system underwent training and evaluation in the context of animal detection, utilizing the widely adopted Microsoft Common Objects in Context (MS COCO) dataset (Lin et al., 2014) for object detection tasks. Within this context, we carefully selected and re-labeled images from the dataset. Images featuring at least one object categorized as an animal were identified as FOI and labeled as 1, while the remaining frames were designated as background and labeled as 0. To align with the scenario, we organized the data in the test set with a specific logic: FOIs and background frames were presented in a fragmented manner, appearing consecutively and alternating with each other. The frames within these fragments were then randomly ordered.

The near-sensor lightweight model was designed to detect and transmit FOIs while effectively filtering out background frames. These detected frames were subsequently transmitted to a server for fine-grained tasks. In all experiments, we used YOLOv5n (Nano) with 32-bit floating-point precision, unless stated otherwise. The entire system was implemented using PyTorch (Paszke et al., 2019).

### 4.2 Online learning

The essence of an adaptive intelligent sensing framework is its ability to enhance the near-sensor model progressively by training it with increasing segments of data over several phases, with each phase's model deployed near the sensor.

**Figure 3:**
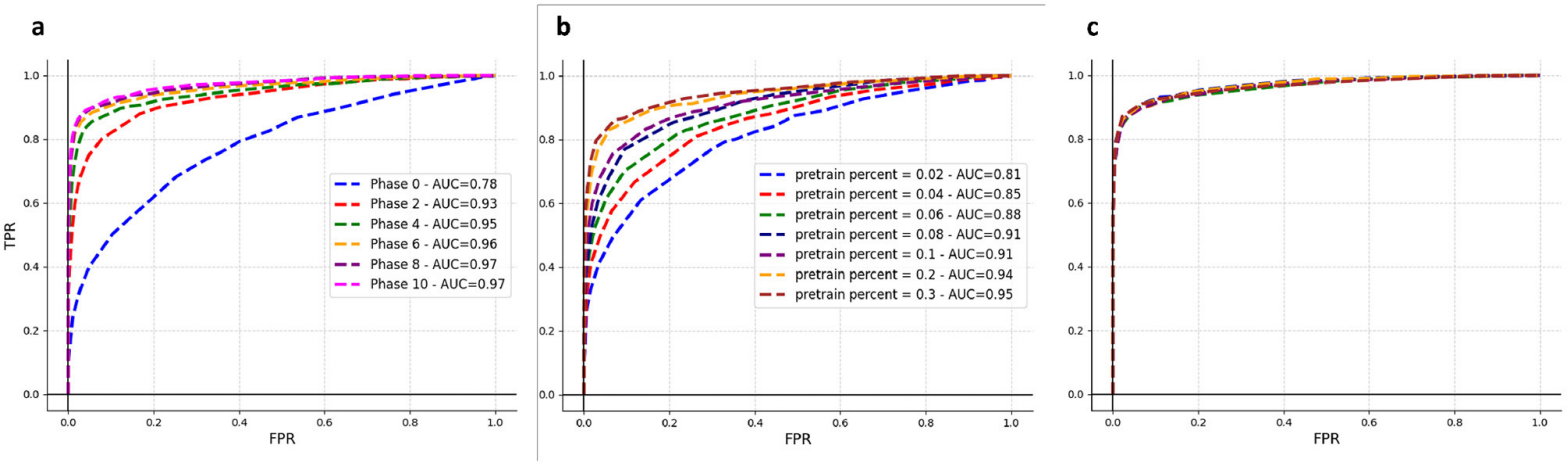
Online learning results. **(A)** Illustrates the training process of a near-sensor model, starting with 2% of the COCO training set for the initial training phase, followed by ten execution phases. **(B)** Each curve represents a near-sensor model upon completing the first training phase, during which a specific percentage of the COCO dataset was utilized, as indicated on the plot. As anticipated, there's a direct correlation between the volume of data utilized in the first training phase and the resultant AUC: the greater the data portion, the higher AUC achieved. **(C)** The same models from **(B)** were subjected to two execution phases using the remaining data, ultimately converging to identical performance levels.

To validate the effectiveness of this strategy in gradually improving the near-sensor model, we initially set up two experiments, outlined as follows. The results of these experiments are illustrated in Figure 3.

In Figure 3A, we start with the assumption that the initial training phase used only 2% of the entire COCO training set, followed by 10 execution phases. The goal for the general intelligent sensing framework is to begin with a model that's slightly better than random, trained in this first phase and deployed near the sensor. By incorporating the misdetections of this initial model into subsequent execution phases, we aim to systematically enhance its accuracy. This iterative process of using the improved model for the next phase leads to steadily increasing AUC values, as shown in Figure 3A. By phase 6, the performance of our near-sensor model almost reaches that of a model trained on the full COCO dataset, despite only using data from the initial training phase and misdetections from subsequent phases. This finding highlights the feasibility of progressively refining the near-sensor model throughout the phases, leveraging the improved model from each previous phase for further enhancements. In real-world sensor deployments, the operational lifespan of the sensor far exceeds the time required for its training. However, our method of online learning ensures the model rapidly adapts to the environment in the early phases of deployment.

From Figure 3B, it becomes evident that initiating the first training phase with varying percentages of the dataset impacts the initial performance of the near-sensor model. Yet, after the model trains on sufficient data (in this case, the whole COCO dataset, presented as two Execution phases), its performance converges to a similar level regardless of the initial starting point, as depicted in Figure 3C. This observation allows deployers to make an informed decision: opting for a more substantial portion of data in the initial phase accelerates the sensor's intelligent capabilities activation but demands increased storage space, as all the training phase data are retained on the server.

In the third experiment, we aimed to explore how well our method could adapt to changes in the data distribution over time, a challenge frequently observed in real-world sensing tasks. Therefore we designed the experiment as follows. Initially, we divided the animal categories in the COCO dataset into two distinct groups. Subsequently, we partitioned the training dataset into two segments, ensuring that the positive samples in the second segment exclusively comprised animals from group 2. This experiment differs from the prior ones due to a shift in the environment, which we modeled by grouping animals, while previously the distribution was consistent across diiferent phases.

**Figure 4:**
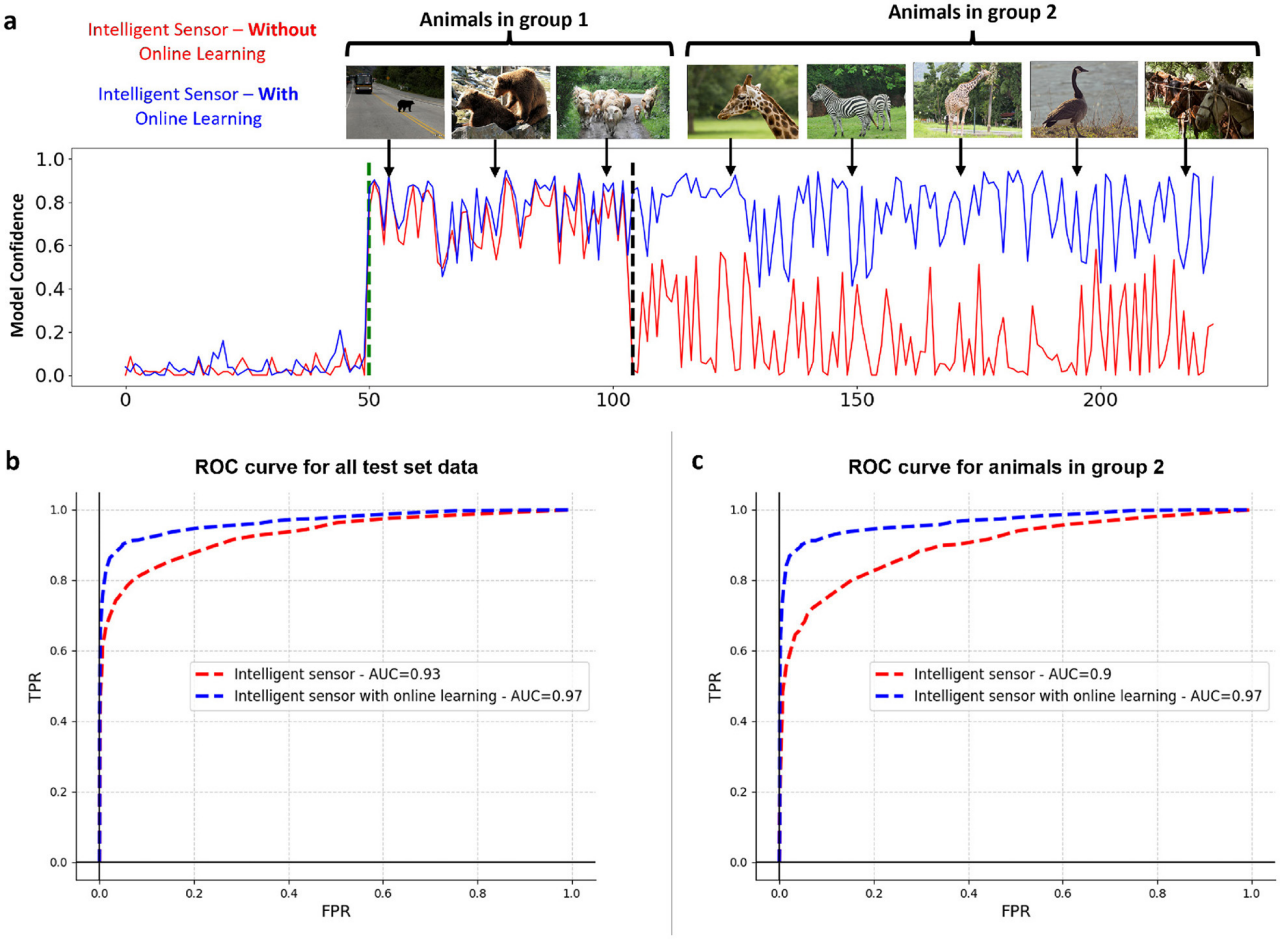
The impact of online learning on the performance of near-sensor model experiencing a shift in data distribution. **(A)** The first model (depicted in red) is only trained on the animals belonging to group 1, but after the change of the environment, the second model (depicted in blue) underwent a training phase to deal with the change of the environment. The plot here shows the confidence of both models in the test environment, where both groups 1 and 2 are present. **(B)** Comparison of ROC curves for two models: the Intelligent Sensing Framework (red) and the Adaptive Intelligent Sensing Framework (blue) across all test data. **(C)** Comparison of ROC curves for the same models: Intelligent Sensing Framework (red) and Adaptive Intelligent Sensing Framework (blue), specifically for test data featuring only animals from Group 2.

We compared the performance of two intelligent sensing frameworks, one with online learning and one without, in a test scenario sequentially featuring animals from two groups. Initially, sensor activity involved only Group 1 animals, followed by the introduction of Group 2 animals. Figure 4A illustrates the comparative performance of these two frameworks in a test setting. The IS/WO (Intelligent Sensing Without Online Learning) framework, only trained on Group 1 animals and lacking an automatic update mechanism, fails to adapt when Group 2 animals are introduced. This results in poor detection of FOIs, leading to their omission on the server side.

On the other hand, the adaptive intelligent sensing framework, designed to accommodate environmental changes through regular retraining, demonstrates robust adaptability. This capability ensures sustained performance levels, enabling accurate FOI detection despite the data distribution shift. This distinction highlights the critical importance of online learning in dynamic sensing environments, where the ability to adapt to new information can significantly enhance detection accuracy and reliability of sensing framework.

To quantify the outcomes, ROC curves for both models across the entire test set, which includes animals from both Group 1 and Group 2, are presented in Figure 4B. Furthermore, Figure 4C specifically illustrates how these models perform on the portion of the test set excluding Group 1 animals, reflectiung the environmental changes.

It's important to highlight that certain general features are common across animals in both groups. For example, most animals in the COCO dataset possess four legs. Consequently, the intelligent sensing framework without online learning might still identify some Group 2 animals based on this generalized feature. However, in real-world scenarios, these broad characteristics may be more challenging for the model to utilize, potentially leading to even lower performance following an environmental shift.

### 4.3 Knowledge distillation

**Figure 5:**
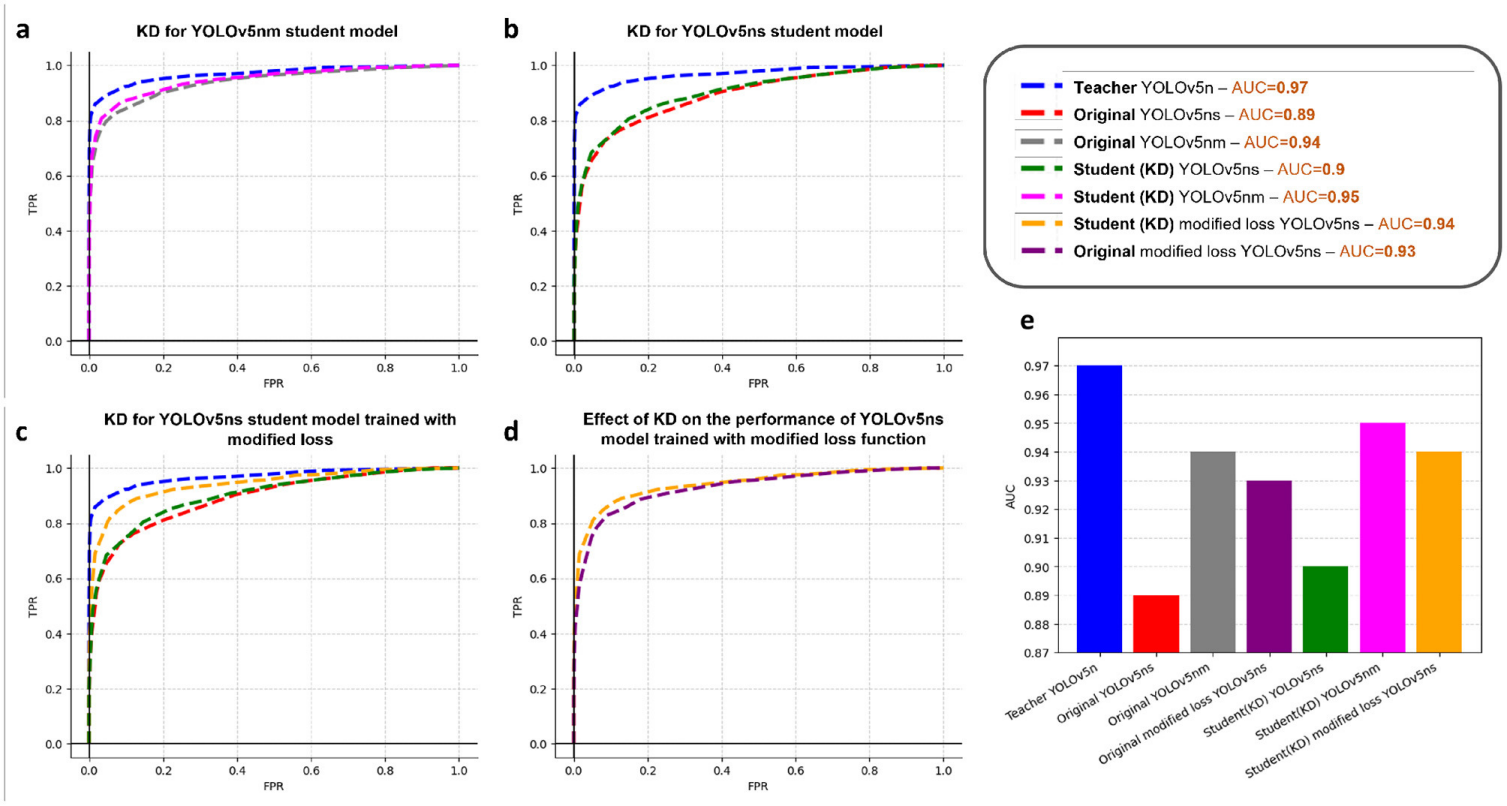
Results of knowledge distillation. **(A)** Distilling knowledge from YOLOv5n (teacher) to YOLOv5nm (student). **(B)** Distilling knowledge from YOLOv5n (teacher) to YOLOv5ns (student). **(C)** Distilling knowledge from YOLOv5n (teacher) to YOLOv5ns with modified loss (student) and comparing with the student model with original loss. **(D)** Comparison of the effect of loss function on the performance of student model. **(E)** Comparison of the performance of teacher and different student models.

In order to show the effectiveness of KD, we introduced a few models, namely yolov5nm (medium, with 24.5% of yolov5n parameter) and yolov5ns (small, with 6.2% of yolov5n parameter), which share the same architecture as yolov5n but vary in the network's depth and the number of filters in different layers (width). The experiment is performed by distilling the knowledge from the largest model (yolov5n) to each of the smaller ones. As illustrated in Figures 5A, B, yolov5nm and yolov5ns trained using distillation from yolov5n, outperform the conventional yolov5nm or yolov5ns, indicating the effectiveness of knowledge distillation to attain a better performance while the size of the near-sensor model is constrained.

Figure 5C demonstrates the benefits of employing the modified loss function, as outlined in the methods section, for training the student model. The orange curve, representing the student model trained with this modified loss function, shows notably superior performance in comparison to the green curve, which depicts the student model trained using the traditional loss function.

Moreover, to confirm that the observed enhancement is attributed to both KD and loss modification, we conducted a comparative analysis. This involved evaluating the performance of the original YOLOv5ns model trained with the modified loss against that of the student YOLOv5ns model trained with both KD and the modified loss function. The results, presented in Figure 5D, validate that the KD-trained model exhibits notable performance gains. Figure 5E provides a comparative analysis of the AUC values for all models trained with KD and modified loss function.

We also investigated the influence of quantization on the model performance. The student model YOLOv5ns trained on the modified loss is quantized into different bit precisions, i.e., 16-bit float point (fp16), 8-bit integer (int8), 5-bit integer (int5), and 4-bit integer (int4). The performance of both the fp16 and int8 quantized models remains unaffected. However, as illustrated by Table 1, when we further reduce bit precision to int5, a slight degradation in AUC is observed (from 0.94 to 0.92), and a degradation in performance is noticeable when the model is quantized to int4 (from 0.94 to 0.89).

**Table 1:** AUC of the student model YOLOv5ns with different quantization levels, trained on the modified loss.

**[-58,10.7]79125.7ptPerformance Quantization**	**fp32**	**fp16**	**int8**	**int5**	**int4**
AUC	0.94	0.94	0.94	0.92	0.89

The terms fp32, fp16, int8, int5, and int4 refer to 32-bit floating point, 16-bit floating point, 8-bit integer, 5-bit integer, and 4-bit integer, respectively.

### 4.4 Energy consumption and data selection

**Figure 6:**
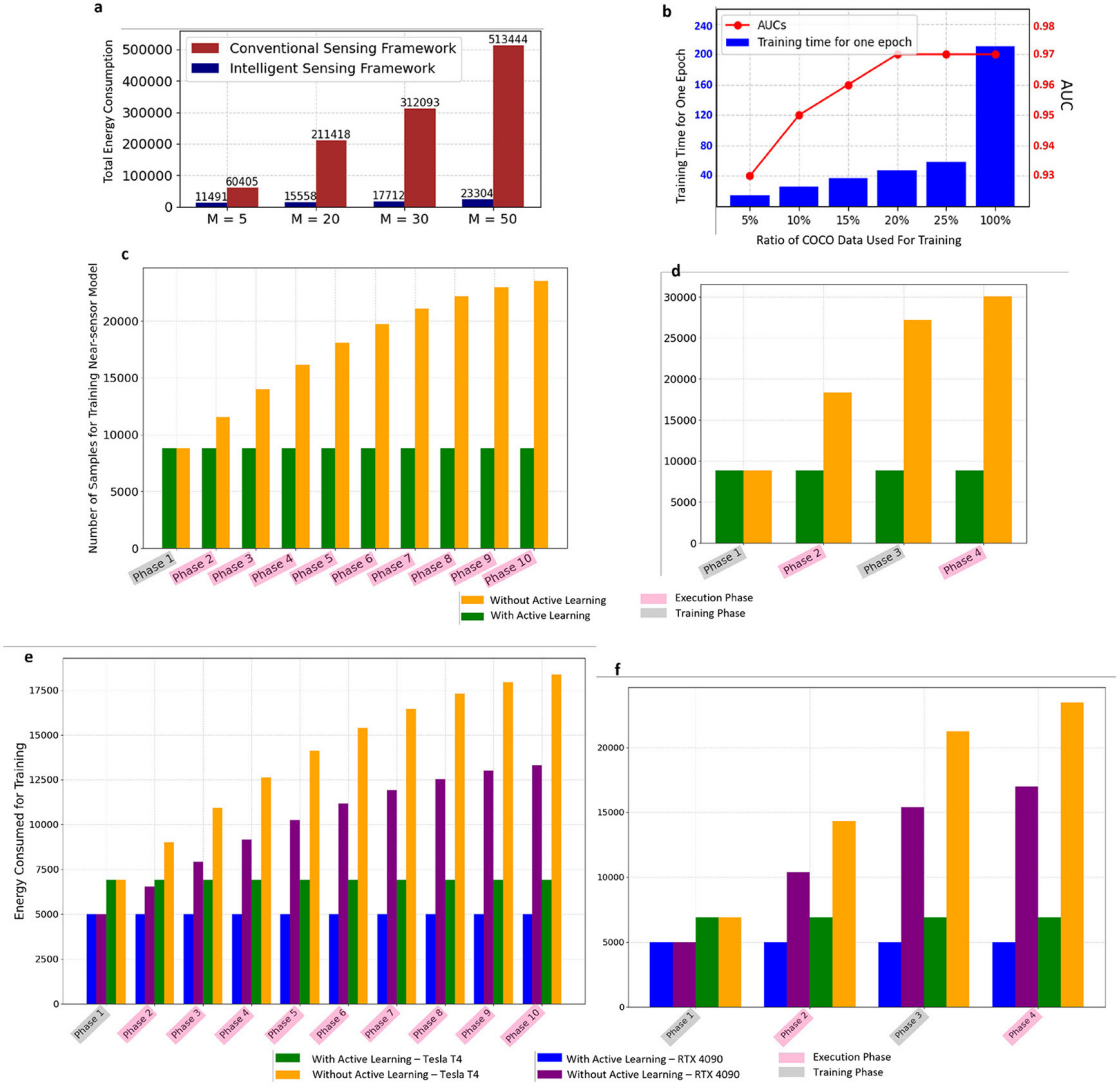
**(A)** Comparison of energy consumption of conventional sensing framework and intelligent sensing framework across different values of *M* (background to FOI ratio). **(B)** The effect of utilizing active-learning-based data selection and fixed size training buffer on training time and model performance. **(C)** Comparison of total number of frames used for retraining the near-sensor model for two frameworks with and without AL. Only the first phase is a training phase and the rest of the phases are execution phases. **(D)** Comparison of total number of frames used for retraining the near-sensor model for two frameworks with and without AL. The first and third phases are training phases, while the second and forth phases are execution phases. **(E)** Comparison of energy consumption for training one epoch with and without AL on two platforms [correspond to **C**] **(F)** Comparison of energy consumption for training one epoch with and without AL on two platforms [correspond to **D**].

The intelligent sensing framework can lead to significant energy and storage savings compared to traditional sensing frameworks, thanks to its selective data transmission policy. If we define *M* as the ratio of the number of background frames to FOIs, the efficiency of the intelligent sensing approach becomes increasingly apparent as *M* rises. This is because the intelligent sensor is capable of filtering out many unnecessary processes on the server side. Figure 6A illustrates these results. In this figure, while the count of FOIs remains constant, altering the number of background frames modifies the *M* ratio. As *M* grows, energy consumption generally climbs due to the higher total number of frames needing processing. However, the intelligent sensing framework demonstrates a significantly smaller increase in energy use compared to conventional sensing frameworks.

In the framework we propose, the most influential training occurs primarily during the first training phase and initial execution phases. After this period, in the absence of environmental changes, our system's energy consumption is similar to that of a typical intelligent sensor. However, our framework does require occasional updates to the near-sensor model at the end of each training or execution phase. The energy consumption and time needed for these updates are crucial factors for us. The training time and energy consumption of deep neural networks are profoundly influenced by the volume of training data. It is in this context that the value of our active learning-based data selection method becomes particularly evident.

As the process progresses, retaining all samples from training phases and misdetections from execution phases results in an increasingly large dataset. Moreover, not all these samples provide valuable information for updating the model. By employing our active-learning-based approach for data selection, which targets filling a training buffer of fixed size, we can significantly lower energy consumption during training without compromising the near-sensor model's performance, as shown in Figure 6B. In our experimental setup, we established a sequence of training and execution phases applied to the entire COCO dataset, with the buffer size determined by the proportion of the COCO dataset utilized in the initial training phase. For instance, if 5% of the COCO dataset was used for initial training, the buffer size for subsequent phases was set to match the number of frames representing 5% of the COCO dataset. While reducing the buffer size and using fewer data points for training and updates may lead to a minimal decrease in AUC, this impact is negligible. Importantly, we observed that, to a certain degree, downsizing the buffer does not detrimentally affect performance. In the subsequent experiment, illustrated by Figures 6C, D, we evaluate the total number of frames utilized for retraining the near-sensor model within two configurations of the adaptive intelligent sensing framework: one employing AL and the other not. Figure 6C shows a setup where only the initial phase is dedicated to training, with subsequent phases being execution phases, each introducing 10% of the COCO training set to the system. Furthermore, Figure 6D adopts an alternating training and execution phase pattern, with each phase presenting 25% of the entire COCO dataset to the framework. As the process advances through the phases, the framework not incorporating AL accumulates significantly more data frames in the training buffer, leading to a marked increase in training time and energy consumption. This escalation is depicted in Figures 6E, F, which show the energy consumption for a single training epoch of the near-sensor model across both frameworks on two distinct platforms (Nvidia RTX 4090 and Tesla T4). This observation underscores the efficiency of our method for sensors with limited resources, achieving substantial reductions in both energy and time required to retrain the near-sensor model. Upon examining Figure 6D and comparing the overhead between phases 2 and 3, it becomes evident that the active-learning-based data selection method is particularly beneficial during a training phase. This efficiency is realized by eliminating some of the samples that the model has already learned well from the training buffer, optimizing the training process.

The modern heterogeneous AI computing system incorporates multiple levels of memory resources. For instance, GPUs have fast SRAM and slow DRAM (or HBM), while FPGAs feature fast on-chip BRAM and slower off-chip DRAM (Dao et al., 2022; Lu et al., 2021). Due to the significant gap in memory access speeds, optimizing training speed necessitates prioritizing access to fast on-chip memory and minimizing communication with slow off-chip memory (Chen et al., 2024). As depicted in Figures 6B, our proposed active-learning method resulted in a reduction of over 80% in training data with < 1% AUC loss. This reduction in training data samples reduces access times to slow off-chip memory by over 80%, thereby decreasing the energy consumption of the training process by ~5 × . We also deployed the model on various edge devices. The measured inference speed and power consumption are recorded in Table 2. We explored three distinct hardware platforms: edge GPU, edge TPU, and edge FPGA. Specifically, for edge GPU, we assessed the Nvidia Jetson Orin (Orin), Jetson Orin Nano (Orin Nano), and Jetson Nano (Nano). Regarding edge TPU, we evaluated the Google TPU USB, TPU Dev Board (TPU Dev), and TPU Dev Board Mini (TPU Mini), utilizing R Pi as the host machine for TPU USB (marked * in Table 2). At the same time, the other TPU development boards possess host CPUs. For edge FPGA, our investigation included the Xilinx ZCU104 and Kria 260. The model kernel for R Pi and Nvidia GPU was implemented using PyTorch. For edge TPU, TensorFlow Lite and PyCoral API were employed for quantization and deployment. As for Xilinx FPGA, Vitis AI was utilized to map the model onto the Xilinx deep processing unit (DPU). Considering the real-time constraint, we believe edge TPU families show more advanced performance than edge GPUs and FPGAs while consuming less power (< 5W), which is ideal for low-power settings. The model's performance on the NVIDIA Jetson series is constrained by PyTorch's optimization for embedded GPUs, in comparison to TPU. The FPGA DPU, on the other hand, has a lower frequency (only 300 MHz) and limited host-kernel bandwidth when compared to TPU platforms (Lee et al., 2023). To achieve better performance on FPGA, customized data path IP and computing unit IP are necessary (Chen et al., 2023).

**Table 2:** Latency and power measurement of the near-sensor model across platforms.

**Platform**	**Orin**	**Orin nano**	**Nano**	**TPU USB**	**TPU Dev**	**TPU Mini**	**ZCU104**	**Kria 260**
Host	Cortex-A78AE	Cortex-A57		Cortex-A72*	Cortex-A53	Cortex-A35	Cortex-A53	
Kernel	GPU			Edge TPU Coprocessor			Xilinx DPU	
Framework	PyTorch			TensorFlow Lite and PyCoral			Vitis AI	
Latency (ms)	30	34	140	7	4	23	56	50
Power (W)	22.9	7.3	3.9	5.02	3.47	0.92	8.9	7.6

The model adopted is YOLOv5n, and * marks utilizing R Pi as the host machine.

## 5 Conclusion

In this study, we have expanded the concept of intelligent sensing by incorporating online learning into the near-sensor model's training process. Our innovative approach eliminates the need for manual labeling across various tasks, allowing for integration with existing sensing frameworks by leveraging server-side model predictions for near-sensor model training. This strategy significantly lowers energy consumption and obviates the need for pre-training the near-sensor model prior to its deployment.

Additionally, our method is adept at adapting to environmental changes through the periodic re-training of the near-sensor model. We achieve this by distinguishing between training and execution phases: during training phases, the near-sensor model processes all data without filtering, ensuring comprehensive training; conversely, in execution phases, it acts as a filter, identifying and discarding non-informative data.

To enhance the efficiency of our sensing framework further, we have employed knowledge distillation. This technique streamlines the near-sensor model, reducing its size while preserving its effectiveness. Moreover, by applying active learning principles, we have minimized the volume of training data required, thereby optimizing the training process. This combination of strategies enhances the practicality and sustainability of intelligent sensing, making it a more viable option for a wide range of applications.

Future research will aim to explore the scalability of our framework, the integration of more sophisticated machine learning algorithms, and the extension of our approach to a wider range of sensing applications.

## Data Availability

The original contributions presented in the study are included in the article/supplementary material, further inquiries can be directed to the corresponding author. The dataset Microsoft COCO object detection for this study can be found in Lin et al. (2014).
